# A neuro-mechanical model containing fast and slow muscle fibres applied to mimic stop and re-start of a stepping leg

**DOI:** 10.1186/1471-2202-14-S1-P86

**Published:** 2013-07-08

**Authors:** Silvia Daun-Gruhn, Ansgar Büschges, Tibor I Toth

**Affiliations:** 1 Emmy Nother Research Group for Computational Biology, Institute of Zoology, University of Cologne, Cologne, D-50674, Germany

## 

The leg muscles of legged animals show differentiation with regard to the contraction velocity of the fibres in them.

Thus one can distinguish between slow, fast and intermediate muscle fibres according to their contraction velocities.

Accordingly, they are innervated by specific motoneurons bearing the corresponding name. The stick insect, because of its relative simplicity, suits particularly well to studying the properties of these muscle types and their neuronal control. Thus a large amount of experimental data is available from this order of animals. We shall therefore refer to them in the following. Bässler et al. [1] showed that the differentiation of muscle fibres implies different functional roles for them. Whereas fast, slow and intermediate muscle fibres contribute to the total muscle force during locomotion, only the slow ones are active in static positions, thus in maintaining the posture of the animal.

To take account of this fact, we extended our existing model [5] by dividing the pairs of the main three antagonistic leg muscles into fast and slow muscle fibres using appropriate anatomical data [2,3]. For the sake of simplicity, we omitted the intermediate ones taking them partly as slow partly as fast muscle fibres. We also included the property of residual stiffness of the slow muscle fibres, which is present even when the innervating motoneurons are silent [4]. It is known from experiments that the residual stiffness is removed by the activity of the common inhibitor motoneuron during locomotion, especially during retraction [6]. This physiological property, too, is part of our extended model. The recruitment of the muscle fibres of the individual muscles was also built in the model, reflecting the recruitment of the innervating motoneurons.

By means of our model, we first demonstrated that locomotion (stepping) is indeed primarily determined by the activity of the fast muscle fibres, even though the slow ones are co-active. We also showed that in static positions the residual stiffness of the slow muscles is capable of maintaining the posture. We then applied our model to elucidate intra-leg coordination of the muscle activity when the animal stops and when it re-starts. According to our model, intra-leg coordination at stopping is brought about by inhibiting the individual motoneurons via inhibitory interneurons, rather than switching off the central pattern generators associated with the antagonistic muscle pairs of the leg. The simulations showed that the leg completes a stance phase after the touch-down of the tarsus until the femur reaches its static horizontal position, and the tibia its static flexion. Depending on the steady-state horizontal position (angle) of the femur, the stepping commenced with a swing or a stance phase. The above simulation results are in good agreement with the relevant experimental findings. The model thus provides a putative intra-leg coordination mechanism that can account for the processes that take place at stop and at re-start of stepping of a multi-joint leg, like that of the stick insect.
